# Initial Testing of a Novel, Mental Imagery‐Based Anxiety Intervention for People With Mild to Moderate Intellectual Disabilities Using a Single Case Experimental Design

**DOI:** 10.1111/jar.70264

**Published:** 2026-06-17

**Authors:** Olivia M. Hewitt, Paul A. Thompson, Craig Steel, Peter E. Langdon

**Affiliations:** ^1^ Intellectual Disabilities Research Institute (IDRIS) University of Birmingham Birmingham UK; ^2^ Oxford Health NHS Foundation Trust, Learning Disability Servcie Slade House, Horspath Driftway Oxford UK; ^3^ Oxford Institute of Clinical Psychology Training and Research Oxford UK

**Keywords:** anxiety, co‐design, intellectual disability, mental imagery, single case experimental design

## Abstract

**Background:**

People with intellectual disabilities experience higher anxiety rates and barriers accessing effective interventions. This study conducted initial testing and refinement of Co‐MAID, a novel mental imagery‐based anxiety intervention co‐designed with people with mild to moderate intellectual disabilities.

**Methods:**

Six participants received 9–12 individual sessions using a non‐concurrent multiple baseline design. Sessions focused on positive imagery generation, attention shifting and image property modification. Acceptability was assessed through interviews and adherence measures.

**Results:**

Five participants completed the intervention with 92.6% session attendance. Template analysis revealed positive experiences with improved mood and reduced anxiety. Three participants (50%) demonstrated reliable change on the primary anxiety measure, meeting clinically significant change criteria.

**Conclusions:**

Co‐MAID demonstrated good acceptability with some preliminary evidence of anxiety reduction in people with mild to moderate intellectual disability. Further research with larger samples and controlled designs will establish the feasibility of a randomised control trial leading to a Phase III trial.

## Lay Summary

- Researchers developed and tested Co‐MAID, a new therapy that uses mental imagery to help people with mild to moderate intellectual disabilities manage their anxiety. The therapy was designed together with people who have intellectual disabilities to make sure it worked for them.
- The study involved six people who attended nine individual therapy sessions with excellent attendance rates (93%). Half of the participants showed meaningful improvements in their anxiety levels and most participants reported feeling better after the sessions.
- This research is important because people with intellectual disabilities often experience higher levels of anxiety but have fewer treatment options available to them. Co‐MAID offers a potentially accessible and effective way to help this population manage anxiety using techniques specifically adapted for their needs.
- This study provides promising early evidence that mental imagery‐based interventions can work for people with intellectual disabilities. The positive results suggest this approach deserves further investigation through larger, more comprehensive clinical trials to see if it could become a widely available treatment option.

## 1 Introduction

### 1.1 Anxiety in People With Intellectual Disabilities

Anxiety disorders are common in people with intellectual disabilities, with prevalence rates being between 4%–17% (Reid et al. 2011). For older people with intellectual disability (aged 55 and over), they are 11 times more likely to have an anxiety disorder diagnosis (this could be any type of anxiety diagnosis) than people with no intellectual disability (El Mrayyan et al. 2019). As with others, people with intellectual disabilities experience various anxiety disorders (e.g., phobias, generalised anxiety disorder, etc.). Anxiety disorders are associated with several adverse outcomes. They are associated with poorer psychological function, social functioning and lower satisfaction regarding daily living (Snoeijen‐Schouwenaars et al. 2019) and with a more sedentary lifestyle and poorer quality of life (Carmeli et al. 2009). People with intellectual disabilities and an anxiety disorder are significantly more likely to experience a range of physical health conditions including thyroid disease, respiratory disease, gastrointestinal disease, arthritis, migraine headaches and allergic conditions (Sareen et al. 2006). Without treatment, they generally persist (Dubé et al. 2024), highlighting the need for effective and accessible interventions for people with intellectual disabilities.

### 1.2 Psychological Approaches to Anxiety in People With Intellectual Disabilities

A range of psychological approaches to anxiety have been developed for people with intellectual disabilities. Langdon et al. (2024) adapted a behavioural intervention for people with moderate to severe intellectual disabilities based on relaxation and graded exposure (The BEAMS‐ID Study). The feasibility of delivering the intervention was tested; it was also found to be acceptable to patients and their supporters, suggesting a definitive trial is warranted.

Patterson et al. (2019) systematically reviewed the literature around the use of third wave therapies (mindfulness‐based approaches, Compassion Focused Therapy, Acceptance and Commitment Therapy and Dialectical Behaviour Therapy) in people with intellectual disabilities. The impact of these therapies on psychological distress or mental health problems was unclear. Whilst a reduction in symptoms of anxiety and depression was found by some studies (e.g., Hall et al. 2013; Hardiman et al. 2018) and reductions in psychological distress were reported by others (e.g., Crossland et al. 2017), some studies found no improvements or a deterioration in mood.

A systematic review of the literature around psychodynamic interventions for individuals with intellectual disabilities was conducted by Shepherd and Beail (2017). Two of the 13 included studies specifically looked at anxiety as a presenting issue (Newman and Beail 2005, 2010). One study found some improvement in functioning (as measured by their ability to assimilate problematic events) (Newman and Beail 2005), whilst the second study found no improvement in functioning post intervention.

### 1.3 Cognitive‐Behavioural Therapy for People With Intellectual Disabilities

Cognitive Behavioural Therapy (CBT) is considered the gold standard intervention for many mental health conditions in the general population, including anxiety disorders (e.g., Stefan et al. 2019; National Institute for Health and Care Excellence 2020) and has been adapted for this clinical group. Whilst people with intellectual disabilities experience higher rates of mental health problems than the general population, they are also thought to be less able to use cognitive techniques in addressing their anxiety. A systematic review of nine studies (Fynn et al. 2023) found that while all studies demonstrated some improvement in symptoms of anxiety in people with mild intellectual disabilities following CBT, significant methodological flaws limit the conclusions that can be drawn regarding the effectiveness of CBT for individuals with intellectual disability. CBT can be difficult for people with intellectual disabilities to engage with for a range of reasons (Fynn et al. 2023). Engaging with metacognitive engaging with metacognitive techniques such as cognitive restructuring and thought replacement remain challenging for this population, possibly due to cognitive inflexibility associated with intellectual disability (Rostambeygi et al. 2021). Studies looking at how to adapt and facilitate these cognitive techniques identify that significant effort is needed in terms of time, rehearsal and modelling, to help those with intellectual disabilities to identify and change their cognition and that even with such adaptations, there remain significant barriers for many people with intellectual disabilities when engaging with these skills (Roberts and Kwan 2018).

### 1.4 Mental Imagery

Mental images are representations of sensory information without a direct external stimulus (Pearson et al. 2015). They occur across sensory modalities (‘seeing with the mind's eye’ or ‘hearing with the mind's ear’ etc.; Kosslyn et al. 2006), can be fleeting or protracted and are positive, neutral or negative in content.

Whilst mental imagery has a clear role in the development and maintenance of various psychological disorders (Hackmann et al. 2011), psychological interventions largely target unhelpful thoughts using language. However, mental imagery elicits emotions more easily and intensely than verbal thoughts through several mechanisms (Pearson et al. 2015; Holmes and Mathews 2010), which suggests various ways of using mental imagery in psychological interventions.

Mental imagery forms an integral part of psychological interventions for mental health problems, including anxiety, in mainstream services, with a robust evidence base (Holmes et al. 2015). Mental imagery‐based interventions to reduce psychological distress focus upon increasing the quantity and quality of positive imagery, shifting attention away from distressing imagery and the use of various metacognitive techniques, such as cognitive restructuring, which includes changing the properties of mental images to demonstrate control over the content and form of imagery. Mental imagery interventions have been successfully adapted for other populations such as children and adolescents (Pile et al. 2021; Schwarz et al. 2020). However, manipulating mental imagery has never been developed as an intervention for people with intellectual disabilities, despite its potential advantages which include being effective, quick and posing less of a cognitive burden than verbal‐based CBT interventions (Blackwell 2019).

### 1.5 Developing a co‐Designed Mental Imagery Anxiety Intervention for People With Mild to Moderate Intellectual Disabilities (Co‐MAID)

Initially, a systematic review of 41 studies was undertaken to establish that people with intellectual disabilities experience mental imagery similar to people without intellectual disabilities (Hewitt et al. 2022). There was evidence to indicate that people with intellectual disabilities experienced rich and vivid mental imagery which they can generate and transform (essential for engaging in mental imagery interventions). As expected, additional prompting and support were needed to elicit mental imagery. This review established that mental imagery interventions had the potential to be accessible for this population. We then explored the phenomenology of mental imagery in people with intellectual disabilities including their ability to control and manipulate mental images, which further supported the acceptability and potential benefit of mental imagery interventions in the population (Hewitt et al. 2023).

Whilst co‐production is best practice to ensure accessibility and acceptability for specific clinical populations, it has rarely been used with people with intellectual disability. Co‐MAID is a manualised mental imagery anxiety intervention that was co‐developed with people with intellectual disabilities who had various additional mental and physical health difficulties (including cerebral palsy, anxiety, epilepsy and autism) and other stakeholders, using a rigorous and iterative methodology, Experience‐Based Co‐Design (Hewitt et al. 2025). The inclusion of three different mental imagery components distinguishes Co‐MAID from other CBT‐based anxiety interventions designed for people with intellectual disabilities.

Co‐MAID comprises 9–12 individual sessions, including rapport building, formulation, psychoeducation, three mental imagery components (generating positive imagery, shifting attention away from distressing imagery and changing the properties of an image) and strategies to help participants retain, practice and generalise skills between sessions and post‐intervention. A workbook for people with intellectual disabilities, therapist manual, treatment protocol and intervention logic model have all been developed. Co‐MAID encourages involving a supporter in the intervention to assist with generalising skills and practising imagery techniques post‐intervention. A supporter can be a family member or paid support worker and involvement may include attending therapy sessions and helping with homework tasks. Whilst having a supporter may facilitate engagement, it is not mandatory, as it may not be appropriate or available to all participants.

### 1.6 Aims

The aim of this study was to model the Co‐MAID intervention in an NHS setting using Single case Experimental Design (SCED). The primary aim was to investigate intervention acceptability. A secondary aim was to capture data using daily mood recordings and standardised anxiety measures and to examine change pre‐ and post‐intervention.

## 2 Method

### 2.1 Design

A non‐concurrent multiple baseline (A_1_BA_2_) case series design was used. A_1_ was the multiple baseline phase. Participants were randomly allocated to either a two, three, or four‐week baseline condition. B was the intervention phase (9–12 sessions of Co‐MAID) and A_2_ was the corresponding two‐, three‐, or four‐week follow‐up period. After the initial three participants were recruited and completed the intervention, the intervention and research methods were refined based upon feedback. Refining the intervention was based on the FRAME expanded framework (Wiltsey Stirman et al. 2019) for reporting adaptations and modifications to evidence‐based interventions. This allowed intervention refinements to be made and documented in a systematic way with a clear evidence trail for each change.

Acceptability was determined using semi‐structured qualitative interviews conducted with participants and supporters 2 weeks after intervention completion. In addition, brief feedback was obtained from participants at the end of each intervention session (see Figure S1). Participants were invited to complete standardised measures both pre‐ and post‐intervention of anxiety, psychological wellbeing and distress and vividness and control of mental imagery. Participants were also asked to provide daily mood ratings.

A favourable ethical opinion was obtained from the West of Scotland Research Ethics Service Committee (REF: 24/WS/0022) along with Health Research Authority (HRA) approval. The protocol was registered (ISRCTN70980810). This study is reported in line with the Single‐Case Reporting guideline In BEhavioural interventions (SCRIBE) (Tate et al. 2016).

### 2.2 Participants

Inclusion criteria were: (1) Diagnosis of mild or moderate intellectual/learning disabilities confirmed through case note review or at screening, (2) Aged 18 years or older, (3) Existing diagnosis of an anxiety disorder confirmed at screening (score of 13 or over on GAS‐ID) and (4) Capacity to provide informed consent.

Exclusion criteria were (1) Currently receiving another psychological therapy for a mental health problem, (2) Presenting with mental health symptoms of a disorder other than anxiety which were judged likely to interfere with their ability to successfully participate in the intervention by the study team (e.g., hallucinations and delusions or suicidality), (3) having a severe or profound intellectual disability. See Figure S2 for details regarding recruitment and participation.

Six participants were recruited from 15 patients screened for participation. Reasons for not participating were: not meeting the inclusion/exclusion criteria (*n* = 4), declining to participate (*n* = 2) and being uncontactable by the researcher (*n* = 3). From the six recruited participants, one discontinued the intervention due to an increase in physical health issues.

Six participants with intellectual disability were recruited through UK learning disability services in one NHS Trust. Five supporters were also recruited. Service waiting lists and new referrals were screened for eligibility by clinicians and those eligible were approached by clinicians. Demographic information for each participant and supporter is provided in Table 1.

**TABLE 1:** Demographic information for each participant and supporter.

	Age	Reason for referral	Ethnicity	Comorbid conditions	Gender	Level of intellectual disability	Accommodation	Relationship to person with ID	Daytime activities
PPT01	36	Anxiety	White British	Fragile X, autism	Male	Moderate	Family home		Attends day services with support
SUP01	69		White British		Female			Mother	
PPT02	60	Anxiety	White British	Diabetes	Male	Mild	Sheltered housing		Attends some community events e.g., Church
SUP02 no supporter available									
PPT03	71	Health anxiety	White British	Diabetes enlarged prostate, bladder stones, hypertension,	Male	Mild	Supported living single occupancy flat		Attends some community events e.g., meeting friends
SUP03	50		White British	Diabetes	Female			Paid support worker	
PPT04	33	Health anxiety	White British	Irritable Bowel Syndrome	Male	Mild	Supported living single occupancy flat		Part time work
SUP04	42		White British		Female			Paid support worker	
PPT05	39	Mixed anxiety and depression	White British	Paranoia/delusions	Male	Mild	Shared lives scheme		
SUP05	48		White British		Female			Shared lives carer	
PPT07	41	Mixed anxiety and depression	White British	Cardiac issues	Male	Mild	Supported living in group home		None
SUP07	56		White British		Female			Paid support worker	

### 2.3 Measures

#### 2.3.1 Glasgow Anxiety Scale for People With an Intellectual Disability (GAS‐ID)

The Glasgow Anxiety Scale GAS‐ID (Mindham and Espie 2003) is a 27‐item self‐rating scale measuring symptoms of anxiety in people with intellectual disability. Responses are provided on a 3‐point Likert scale of ‘no, sometimes’ or ‘a lot’. Total scores range from 0 to 45, with a higher score indicating higher anxiety. Scores of 13 or over indicate an anxiety disorder. The GAS‐ID has good test–retest reliability, internal consistency and concurrent validity (Mindham and Espie 2003). This was used as the primary outcome measure.

#### 2.3.2 Adapted Patient Health Questionnaire (PHQ‐9)

The PHQ‐9 is a 9‐item questionnaire used to assess the severity of symptoms of depression over the past 2 weeks (Kroenke et al. 2001) in the general population. This has been adapted for use in people with intellectual disabilities (Jenkins et al. 2025), showing good reliability and validity. It consists of 9 items and responses are provided on a 4‐point Likert scale ranging from ‘no days’ to ‘nearly every day’. Each item is presented in an easier read format (large text, visual representation of the Likert scale accompanies each item) and a picture illustrating the meaning of that item.

#### 2.3.3 Adapted Generalised Anxiety Disorder (GAD‐7)

Generalised Anxiety Disorder (GAD‐7) (Spitzer et al. 2006) is a seven‐item questionnaire designed to assess levels of generalised anxiety in the general population. It has good levels of validity and reliability and can be used as a self‐report or interview administered tool. An adapted version of the GAD‐7 has been developed for use with people with intellectual disability (Jenkins et al. 2025) and shows good reliability and validity in this population. Responses are provided on a 4‐point Likert scale ranging from ‘no days’ to ‘nearly every day’. Each item is presented in easier read format (large text, visual representation of Likert scale accompanies each item) and a picture illustrating the meaning of that item.

#### 2.3.4 Psychological Therapies Outcome Scales—Intellectual Disabilities (PTOS‐ID)

The Psychological Therapies Outcome Scales—Intellectual Disabilities (PTOS‐ID; Vlissiles et al. 2017) is a psychometrically robust, three‐factor therapy outcome measure designed for use with people with intellectual disabilities. It measures overall positive psychological wellbeing and psychological distress (comprising two factors ‘anger and mood’ and ‘anxiety’). Higher scores on the positive wellbeing index indicate higher levels of wellbeing and vice versa on the psychological distress index.

#### 2.3.5 Vividness of Visual Imagery Questionnaire (VVIQ)

The Vividness of Visual Imagery Questionnaire (Marks 1973) consists of 16 items in four groups of four items. Participants consider four different mental images prompted by the interviewer and assess the vividness of aspects of this image on a 5‐point Likert scale. To improve accessibility of this measure, the wording used to describe the task was simplified and the interviewer was able to provide additional scaffolding and prompts for participants when completing this measure.

#### 2.3.6 Mental Imagery Questionnaire for People With Intellectual Disability (MIQ‐ID)

The Mental Imagery Questionnaire for People with intellectual disability (MIQ‐ID) (Hewitt et al., manuscript in preparation) is an eight‐item questionnaire, with responses of ‘yes’, ‘sometimes’ and ‘no’ provided on a 3‐point Likert scale. It has been adapted from two subscales of the Negative Mental Imagery Questionnaire (MIQ‐N; Oaie et al. 2025), concerning realness and controllability of mental imagery. Psychometric testing of this measure is being conducted.

Standardised outcome measures (GAS‐ID, PHQ‐9, GAD‐7, PTOS and either VVIQ or MIQ‐ID) were administered at baseline, mid intervention, end of intervention and at 2 week follow up.

#### 2.3.7 Daily Mood Recordings

Daily records of mood on a Likert scale of 1–5 (with corresponding qualitative descriptors) were completed either electronically through the Daylio app or a pen and paper version of these rating scales. For this scale, scores of 1 corresponded to a descriptor of their mood that day as ‘awful’, 2 corresponded to ‘bad’, 3 corresponded to ‘OK’, 4 corresponded to ‘good’ and 5 corresponded to ‘great’. Each score was visually represented by a different coloured image of a face with corresponding facial expressions. For participants using the app, weekly summary reports of daily scores could be emailed to the research team. The Daylio app allows for a daily prompt to be sent to participants to remind them to complete their recording for that day. Four participants recorded their mood independently and two with assistance from their supporter. Four participants recorded their daily mood through the Daylio app and two used a paper and pen version.

#### 2.3.8 Safety

Safety was evaluated by monitoring and analysing adverse events to determine their severity, expectancy and relationship to study procedures (Grant et al. 2018).

#### 2.3.9 Intervention

The Co‐MAID intervention was delivered to participants by one therapist (OH) via a participant workbook and therapist manual to enhance procedural fidelity. The participant workbook contained easy read and accessible materials to support the delivery of the intervention. Intervention sessions for five participants were delivered at their home address, with PPT05 receiving the intervention at their local NHS mental health clinic. Aspects of Compassion Focused Therapy are incorporated in the intervention and include developing self‐soothing strategies such as grounding and breathing exercises at the start of each therapy session. The main target of the intervention is to develop mental imagery skills, such as generating positive images and to practice changing aspects of an image to develop metacognitive skills. Examples of changing aspects of an image included perceptual changes (e.g., changing the colours, light, size of the image), behavioural changes (e.g., changing what they did) or relational (e.g., adding someone else into the image). Table 2 summarises the focus of each intervention session.

**TABLE 2:** Summary of content for intervention sessions.

Baseline assessment	Complete pre‐intervention measures and confirm informed consent. − Discuss role of supporter, any limits the participant wishes to put on their involvement, and who might be best placed to support. − Opportunity to build rapport and answer any questions. − Explain therapist's role and that of research assistants. − Introduction to the intervention.
Intervention session 1	Understand the development and presentation of anxiety for the participant. − Learn about anxiety and how this is experienced in our bodies. Understand that anxiety is a feeling and that one of the things that maintains anxiety is the thoughts we have. Thoughts can be verbal or visual. − Collaboratively develop formulation about the development and maintenance of anxiety, with emphasis on role of mental imagery as maintaining factor − Focus on rapport building.
Intervention session 2	Psychoeducation about anxiety and mental imagery − Learn about mental imagery. Discuss verbal thoughts vs. mental imagery in relation to emotions. − Use examples to explain the power of mental images vs. real situations. This could include participants hearing real music vs. imagining hearing music. − Learn that having control over our images can reduce the distress caused by the images. − Using positive images to evoke happy and safe feelings.
Intervention session 3	Understanding and practicing mental imagery − Purpose is to help participant understand what we mean by a mental image and to practice their mental imagery skills in terms of generating and manipulating mental images across different sensory modalities. − To continue to explore how mental imagery manipulation can impact on how they feel in their body and their mood. − Identifying an image to work with. To collaboratively explore person's ability to generate mental image and get a full description of the image
Intervention session 4	Mental imagery technique: Generating positive image exercises. − Use the calm place and kind helper exercises. − Agree on the rationale for using positive imagery—could be to increase feelings of calm. − Link the image to a cue e.g., associate image with a smell to help recall. − Audio record the exercise so client can listen back later. − Make a plan about how the images will be practiced in everyday life − When/to what extent the supporter can be involved with this process between sessions.
Intervention session 5	Mental Imagery technique: Attention switching exercise. − Participant switches focus of attention from internal image to outside world. − Bring to mind a mildly troubling image. What can you see/smell/hear/taste etc.? − Check what emotions this provokes and how strong they are (can use visual analogue scale here to help with this) − Shift the participant's focus. Can be done using objects in the room or though looking at a range of additional objects provided by the therapist. Do this for 1 min − Now bring the focus back to yourself. How do you feel now? What has happened to [emotion]. How strong is it on scale of 1–10? − Identify any ways in which the image and associated emotions have changed. Can ask the participant how or why they think these changes have come about.
Intervention session 6	Mental imagery technique: Changing the properties of an image. − Explain rationale for this exercise using clip from Harry Potter and the Prisoner of Azkaban film. − Purpose of exercise is to change the properties of the image. This allows the participant to gain control over the image and to understand that images can be changed and are not reflections of reality per se. − To identify an image to work with. To help the participant identify the emotion it elicits and how strongly this is experienced. − Examples of imagery manipulation techniques include imagining changing the colour of the image or imagining popping the image like a balloon. − To support the participant to experiment with changing different properties of the image. − To revisit the image and identify the emotion it elicits and how strongly this is experienced. − To explore what the participant has leant from this exercise/what it means in terms of the reality and controllability of mental imagery
Intervention session 7	Develop Blueprint (possible joint session with supporter) − To work with participant to develop an accessible summary of the work completed. − What did you learn in our sessions together? − How will you practice these new skills? − What will you do to remember to be kind to yourself in the future? − If you were feeling anxious or having tricky images, what would you want to remind yourself of/say to yourself? − If you were giving advice to someone who also had anxious pictures, like you, what would you say to them to help them feel things were more manageable?
Intervention session 8	Review blueprint, complete post intervention outcome measures (possible joint session with supporter) − To recap blueprint and share with supporter. − To reflect on the work undertaken and how this can be continued/maintained. − To understand what the participant found to be the most powerful/helpful mental imagery strategies. − To discuss plan for the future e.g., accessing additional psychological support.

### 2.4 Procedure

All therapy sessions were completed by OH. Additional research procedures such as gaining informed consent and conducting parts of process evaluation were conducted by a research nurse from the Trust R and D department.

At initial assessment participants completed the GAS‐ID, PHQ‐9, GAD‐7, PTOS and either VVIQ or MIQ‐ID. Participants were then randomised into baseline times of varying length which was decided by a computer programme (https://www.randomizer.org/) PPT03 and PPT07 were allocated to a 2 week baseline, PPT01 and PPT02 to a 3 week baseline and PPT04 and PPT05 to a 4 week baseline. Participants completed the daily mood recordings from assessment until the end of their follow up period (2, 3 or 4 weeks after their final intervention session). No blinding or masking was used. Brief session by session feedback was obtained for each participant, who rated the session ‘good’, ‘OK’ or ‘bad’ and provided brief feedback on helpful and unhelpful aspects of the therapy session.

Semi‐structured qualitative interviews were conducted with participants and their supporter at 2 week follow up, by a research nurse who had not been involved in delivering the intervention.

### 2.5 Analysis

Semi structured qualitative interviews with participants (*n* = 5) and supporters (*n* = 3) were conducted at 2 week follow up. Data was analysed using Template Analysis (Brooks et al. 2015). The following steps were undertaken:

1. OH became familiar with the data through repeated reading of transcripts and listening to recordings of the interviews.
2. Preliminary coding of the data was conducted. A priori themes were identified in advance through consultation with the literature and discussion within the research teams as likely to be helpful and relevant to the analysis. These were tentative themes which could be removed or redefined if not useful to the analysis.
3. Emerging themes were organised into meaningful clusters, with relationships between groupings being defined.
4. An initial coding template was developed on the basis of two participants and one supporter interviews.
5. The initial template was applied to the remaining transcripts and modified to accommodate additional themes.

Mood, anxiety symptoms, psychological wellbeing, psychological distress and mental imagery were evaluated through inspection of descriptive data. Measures of daily mood recordings were entered and graphed using Microsoft Excel. Monitoring phases were separated into baseline (all recordings prior to first intervention session), Intervention (up to the day of last intervention session) and follow up (all recording subsequent to last intervention session). Visual analysis was conducted looking for change in central tendency, trend, or variability, in accordance with visual analysis guidelines (Kazdin 2020). Statistical analysis of daily mood ratings was conducted using TAU‐BC (baseline corrected TAU) calculated to compare each condition (baseline, intervention, follow up) for each participant through a single‐case effect size calculator (Pustejovsky et al. 2024). TAU‐BC assesses for the presence of monotonic trend in baseline and corrects it, if needed (Tarlow 2017). Three comparisons were conducted: baseline versus therapy (to control for the effects of time and monitoring); therapy versus follow‐up; and finally baseline and therapy were also combined and compared to follow‐up to comprehensively evaluate post‐therapy effects compared to the preceding phases. Reliable (RCI) and clinically significant change (CSC; Jacobson and Truax 1991) were calculated using the Leeds Reliable Change Index Calculator for a single case (Morley and Dowzer 2014) for the GAS‐ID. RCI & CSC were calculated using clinical and non‐clinical sample means and standard deviations from previous research (Mindham and Espie 2003). CSC was calculated using a cut off score of 13.

## 3 Results

### 3.1 Adaptations to Intervention

A total of 34 intervention adaptations were collated from feedback from participants, their supporters, the therapist delivering the intervention, NHS service manager and academic supervisors (see Table S1). Eleven proposed amendments were declined and the remaining 23 were implemented. Examples of proposed changes that were declined included changes to a single word on the information sheet which would have required ethical review and reducing the amount of text on one page of the participant's workbook which was considered appropriate in the level of detail provided. Several proposed changes were agreed to be made for a future feasibility phase of testing the intervention but were not required during this initial testing phase. The most significant change made was to replace the VVIQ with the MIQ‐ID. This was made as participants found the VVIQ burdensome and repetitive. The MIQ‐ID has been devised to target pertinent aspects of mental imagery that will be targeted by the Co‐MAID intervention (i.e., the control participants feel over their imagery and the vividness or realness of the images they experience). There were no significant deviations to the protocol in terms of time spent in baseline, treatment or follow up.

### 3.2 Acceptability

#### 3.2.1 Dropout Rate

One participant (PPT03) dropped out of the intervention after session five. This was due to an increase in physical health appointments for multiple comorbid conditions, which made attending psychology sessions unfeasible.

#### 3.2.2 Adherence

All five participants who completed the intervention attended all nine sessions, giving an overall session attendance rate of 92.6%.

#### 3.2.3 Safety

No serious adverse events were reported.

### 3.3 Completion of Standardised Measures

Completion rates for the GAS‐ID, PHQ‐9, GAD‐7 and PTOS‐ID were all 100% at baseline, mid intervention, end of intervention and follow up with no missing data.

The VVIQ was administered to PPT01, PPT02 and PPT03 and replaced with the MIQ‐ID after the intervention refinement process. This was due to high levels of missing data (average 75% of items not completed) for the VVIQ.

The MIQ‐ID replaced the VVIQ and was administered to PPT04, PPT05 and PPT07 and completion rates were 100% with no missing data.

### 3.4 Completion of Daily Mood Ratings

PPT01 completed 99.1% of daily mood recordings. This number was 97.0% for PPT02, 83.6% for PPT03, 100% for PPT04, 99.1% for PPT05 and 55% for PPT07.

### 3.5 Feedback Regarding Aspects of the Intervention

In session 9 of the intervention, participants were asked to rate 18 different aspects of the intervention (see Figure S3). Seventeen percent of this data was missing, partly due to some items not being applicable for the participant (e.g., getting to know the supporter better not applicable for PPT02). Additional feedback identified aspects of the intervention that were experienced as helpful, such as using an app to practice breathing exercises regularly and acquiring new skills around imagery and how flexibly these could be used. Three negative aspects of the intervention were identified: (1) needing to complete feedback forms regularly was mentioned by two participants, (2) having one between‐session task that reminded one participant of a homework sheet from school (which they did not enjoy completing) and (3) one participant wanted more intervention sessions.

### 3.6 Semi Structured Qualitative Interviews

Seven superordinate themes were identified from interviews with participants and supporters.

#### 3.6.1 Theme 1: Overall, Participants Thought the Intervention Improved Mood and Functioning

Three participants and three supporters felt the intervention decreased anxiety and was helpful. Participants were described as less anxious with better overall mood. PPT05 was described as engaging more with daily life and less isolated:

he's gone back to the [person] he used to be… his mood, his engagement with us. He's wanting to go out and do things is… absolutely it's amazing because we were really worried. (SUP05).

PPT02 did not think the intervention helped their mood but enjoyed the sessions.

#### 3.6.2 Theme 2: Having a Supporter Take Part in the Intervention

Only PPT02 did not have a supporter and felt it would have been helpful for understanding content. Supporter roles included reminding participants of session timings, transportation, attending sessions, helping with between‐session tasks and sending daily recordings to researchers.

Several participants and supporters noted positive impacts on their relationship. Attending sessions allowed supporters to see how participants navigated new experiences. The therapy created shared goals:

it strengthened our relationship again because we had sort of lost our way. (SUP05).

Supporters learned new skills that benefitted them personally and gained better understanding of anxiety's impact on the person they support.

I would just became more aware of, being mindful of him and what was going on with him. (SUP05).

#### 3.6.3 Theme 3: Helpful and Unhelpful Aspects of Intervention Format

Some appreciated sessions at home in familiar environments, while others preferred neutral spaces. Individual sessions were beneficial, especially for participants unable to engage with group formats.

Supporters highlighted the importance of flexible, person‐centred approaches, allowing irrelevant sections to be skipped while focusing on helpful elements.

That's what you kind of need, not ‘This is the programme, and we're set in stone’ here. (SUP04).

Participants valued certificates acknowledging their completion. PPT02 framed his certificate.

#### 3.6.4 Theme 4: Experience of Different Intervention Components

Understanding anxiety and its effects was helpful but challenging for some participants:

Talking about anxiety was hard to do It's a bit hard, bit hard. (PPT01).

Sessions began with 5–10 min of body scans, breathing exercises and grounding with using essential oils. These were experienced as beneficial:

he really liked the beginning… He had a lavender smell, and you know, and [therapist]'s calm voice… I think he really benefited from all those kind of things. (SUP04).

Mental imagery components were widely reported as easy to engage with. Participants found generating positive images helpful and accessible:

He preferred the meditation [calm place exercise]. He definitely liked that kind of thing. (SUP05).

Some participants found specific between session tasks helpful, though preferences varied (e.g., breathing apps were liked, written homework disliked by PPT04).

#### 3.6.5 Theme 5: Generalising New Skills

Remembering to use skills was challenging for participants, though supporters noted some implementation of techniques in daily life. Having a blueprint to recap and capture intervention information helped retain new skills. Both participants and supporters raised concerns about motivation impacting ability to continue practising skills independently:

I think he does remember them [mental imagery skills] and hopefully he will actually use them. (SUP01).

#### 3.6.6 Theme 6: Providing Information to the Study Team

Apps for data collection were helpful, allowing some participants to work independently. Despite five measures at four timepoints, participants weren't overly concerned and mentioned benefits of completing questionnaires. Some participants enjoyed regular mood tracking and found it helped identify improvements. Two said they would continue daily mood logging after research ended.

#### 3.6.7 Theme 7: An Accessible Intervention

Participants and supporters praised the therapist's friendly, engaging approach and ability to explain concepts accessibly for people with learning disabilities.

Accessible materials included video clips, images with text and audio recordings. Participants enjoyed making video blueprints, emphasising the collaborative nature.

Apps collected mood data and provided tailored breathing exercises. Participants valued the independence this allowed and this suggests potential for exploring the use of apps within interventions.

### 3.7 Daily Mood Recordings

**FIGURE 1:**
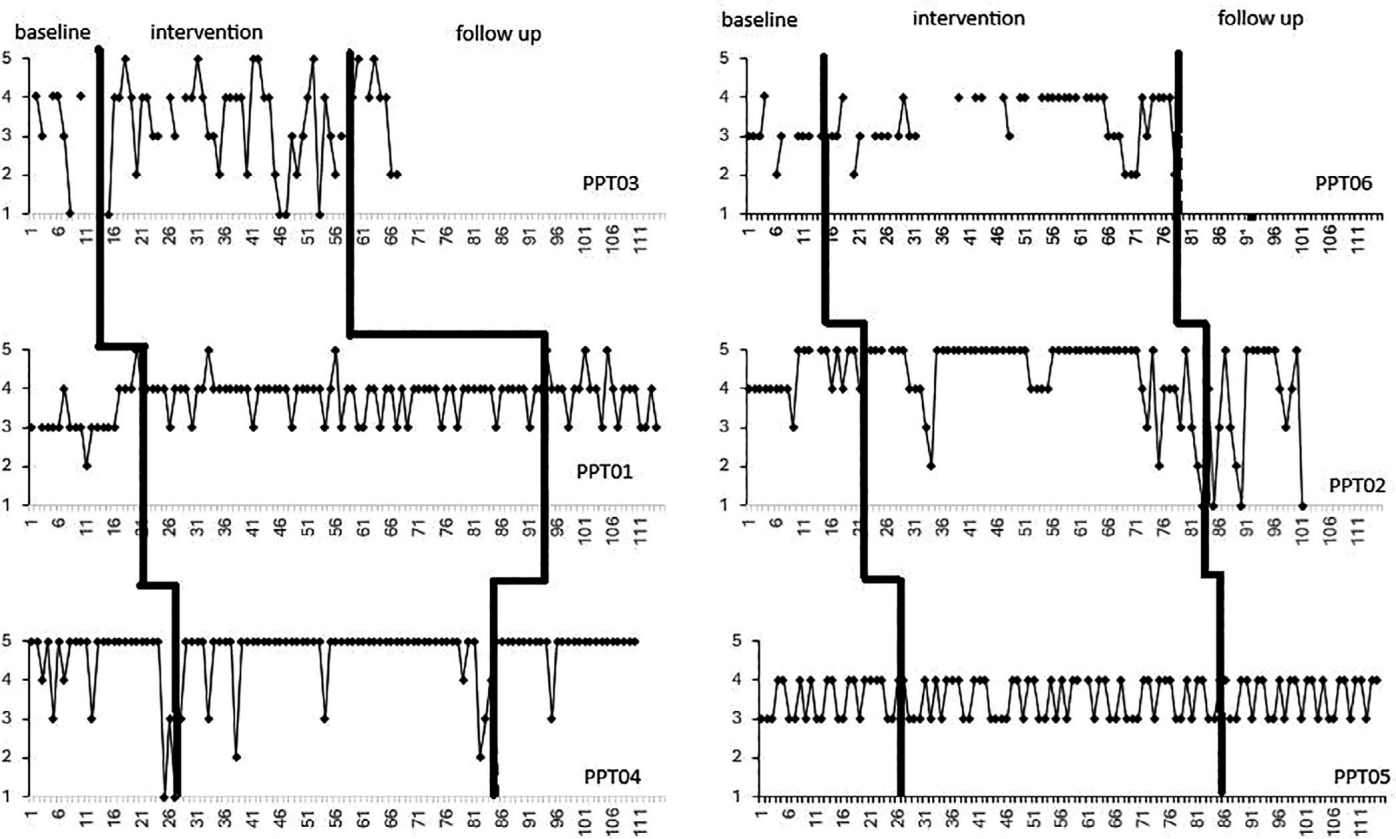
Daily ratings of mood.

Visual representations of daily mood recordings for participants are displayed in Figure 1.

Results were analysed using baseline corrected Tau (see Figure S4).

Daily mood recordings showed a small effect between baseline and intervention for participant one (τ‐BC = 0.47, 95% CI [0.19, 0.67]) and these changes were maintained at follow up (τ‐BC = 0.44, 95% CI [0.08–0.68]). Participant 2 showed a dip in mood during intervention associated with the illness of his brother (τ‐BC = 0.22, 95% CI [−0.08–0.47]). He then showed more variability in his mood towards the end of the intervention when he disclosed a historical attack which was associated with symptoms of Post Traumatic Stress Disorder (PTSD). This mood variability continued over follow up as he waited for an evidence‐based intervention for PTSD (τ‐BC = −0.30, 95% CI [−0.55–0.01]). Participant 3 showed mood variability largely in line with his multiple physical health conditions which continued until he dropped out of the intervention (τ‐BC = 0.03, 95% CI [−0.39–0.44]). Participant 4 may have experienced a ceiling effect especially during intervention and follow up phases (τ‐BC = 0.10, 95% CI [−0.09–0.49]). Participant 5 reported largely stable mood which showed little change across phases (τ‐BC = 0.09, 95% CI [−0.21–0.37]). The daily mood measure may have been insufficiently fine to capture changes in his mood from day to day. Participant 6 found remembering to record his mood more challenging, which improved when the daily reminder was changed to mid‐day (rather than in the evening) at the suggestion of his supporter. A dip in his mood at the end of intervention was associated with his anxiety about a reduction in support (τ‐BC = 0.39, 95% CI [−0.01–0.67]). Unfortunately, this participant did not understand the need to continue recording mood over follow up period, so no data were available.

### 3.8 Reliable and Clinically Significant Change

Results for standardised assessments are provided in Table 3. At end of intervention, three participants (50%) reported reliable change on the GAS‐ID. All three also met criteria for clinically significant change (PPT01, PPT04 and PPT05). One Participant showed no reliable change (PPT02) and data was missing for one participant (PPT03).

**TABLE 3:** Results of standardised measures.

Measure	PPT01				PPT02				PPT03				PPT04				PPT05				PPT06			
	Baseline	Mid	End	Follow‐up	Baseline	Mid	End	Follow‐up	Baseline	Mid	End	Follow‐up	Baseline	Mid	End	Follow‐up	Baseline	Mid	End	Follow‐up	Baseline	Mid	End	Follow‐up
GAS‐ID	19	15	12	16	27	29	24	25	19	14	—	—	22	15	12	13	19	9	4	2	13	9	9	10
PHQ‐9	7	6	6	6	17	21	20	18	3	5	—	—	4	2	2	0	11	2	1	0	8	4	8	4
GAD‐7	3	3	2	3	13	16	15	20	4	7	—	—	1	10	0	2	11	0	0	0	3	4	7	8
PTOS‐ID Distress	29	27	8	8	22	27	34	22	29	16	—	—	8	15	10	9	14	2	1	0	9	10	19	14
PTOS‐ID Wellbeing	20	22	24	20	30	30	29	28	27	23	—	—	26	30	33	32	22	31	29	30	20	21	21	22
VVIQ	12	—	28	27	58	—	—	—	—	—	—	—	—	—	—	—	—	—	—	—	—	—	—	—
MIQ‐ID	—	—	—	—	—	—	—	—	—	—	—	—	—	15	15	15	—	12	18	19	9	6	12	7

*Note: —*, missing data.

Abbreviations: GAD‐7, Adapted Generalised Anxiety Disorder scale; GAS‐ID, Glasgow Anxiety Scale for People with an Intellectual Disability; MIQ‐ID, Mental Imagery Questionnaire for People with Intellectual Disability (PPT04–PPT06 only); PHQ‐9, Adapted Patient Health Questionnaire; PTOS‐ID Distress, Psychological Therapies Outcome Scales—ID (psychological distress scale); PTOS‐ID Wellbeing, Psychological Therapies Outcome Scales—ID (psychological wellbeing scale); VVIQ, Vividness of Visual Imagery Questionnaire (PPT01–PPT03 only).

## 4 Discussion

This study provides initial evidence supporting the acceptability of Co‐MAID, a co‐designed mental imagery‐based anxiety intervention for people with mild to moderate intellectual disabilities. The findings demonstrated that mental imagery techniques can be successfully adapted and implemented with this population, addressing a significant gap in accessible psychological interventions.

The primary aim of the study was to investigate intervention acceptability. Participants rated the therapy as highly acceptable and with one exception, completed all intervention sessions. Supporters described the intervention as bespoke for people with intellectual disabilities, flexible (and thus able to focus on the most pertinent topics for each participant) and engaging. Participants described enjoying therapy sessions, benefiting from a better understanding of their anxiety and finding exercises around self‐soothing (e.g., breathing) and amending mental imagery to be helpful and easy to engage with. There were no serious adverse events. The high session attendance rate (92.6%) and excellent completion rates for standardised measures (100%) indicate strong acceptability of the intervention format and procedures. This is particularly encouraging given the challenges often faced in engaging people with intellectual disabilities in psychological interventions. The qualitative findings further support acceptability, with participants and supporters reporting positive experiences and meaningful improvements in mood and functioning.

After the intervention was completed with the first three participants, it was reviewed and refined and a number of adaptations implemented. Details of all proposed intervention refinements are provided in Table S1.

The secondary aim was to capture data using daily mood recordings and standardised anxiety measures and to examine change pre‐ and post‐intervention. While completion rates for both daily mood recordings and standardised measures were high, daily mood ratings showed significant changes in self‐reported daily mood ratings for only one participant (PPT01). The varied patterns in daily mood recordings highlight the complexity of measuring change in this population. While some participants showed sustained improvements, others experienced mood variability related to external factors such as physical health conditions or disclosing historic traumatic events. This underscores the importance of considering individual circumstances and using multiple outcome measures when evaluating interventions for people with intellectual disabilities. Half of the participants who completed the intervention demonstrated reliable and clinically significant change on the primary outcome measure (GAS‐ID). This suggests that the intervention may have been associated with positive changes. This is notable given the brief nature of the intervention (all participants completing the intervention within 9 sessions) compared to traditional CBT approaches that often require more extensive adaptation and longer treatment periods for people with intellectual disabilities.

### 4.1 Limitations

Several limitations should be acknowledged. The small sample size (*n* = 6) and single case experimental design, while appropriate for initial testing, limit generalisability of findings and the conclusions that can be drawn about causality. Whilst the use of randomisation and multiple baselines increased the robustness of the SCED methodology, this type of design and the loss of a single participant limit the strength of the conclusions that can be drawn. Additionally, the reliance on self‐report measures may be problematic for some participants with intellectual disabilities, despite the use of adapted versions. Three of the participants and two supporters taking part in the semi‐structured interviews had received a refined version of the intervention.

The daily mood recordings showed considerable individual variation in completion rates and interpretability. Additionally, daily mood recordings (Figure 1) do not show a clear impact of the intervention, due to a combination of factors including ceiling effect on the daily measures and difficulties in obtaining accurate daily measures of mood and anxiety in this population. This outcome measure requires further refinement if it were to be used in the future. The lack of follow‐up data for one participant and the brief follow‐up period (2 weeks) also limited our understanding of longer‐term effects.

### 4.2 Clinical Implications

Clinically Co‐MAID offers several advantages over traditional verbal‐based CBT approaches. Mental imagery techniques appear to pose less cognitive burden while remaining engaging and accessible. The incorporation of multi‐sensory elements, visual materials and technology (such as breathing apps) enhanced accessibility and independence for participants.

The role of supporters emerged as particularly important, with qualitative findings highlighting benefits not only for participants but also for supporter relationships and understanding. This suggests that involving supporters may be a key component for maximising intervention effectiveness and skill generalisation.

The findings suggest several considerations for clinical practice. Developing therapist experience and competence in using mental imagery‐based interventions for this population may be of value. How best to incorporate supporter involvement while maintaining appropriate boundaries and consent processes requires consideration.

The accessibility features that contributed to success—visual materials, multi‐sensory approaches, flexible pacing and person‐centred adaptation—should be standard elements of implementation. Co‐MAID challenges previous assumptions about cognitive limitations and highlights the potential for innovative approaches that build on strengths rather than focusing on deficits. Services may wish to explore integrating brief mental imagery techniques into existing care pathways for people with intellectual disabilities experiencing anxiety.

### 4.3 Theoretical Implications

The success of mental imagery techniques in this population has important theoretical implications. The findings support previous research suggesting that people with intellectual disabilities can engage with and manipulate mental imagery when provided with appropriate support and scaffolding. This challenges assumptions about cognitive limitations and opens new avenues for psychological intervention development. However, measuring changes in the vividness and control of mental imagery was difficult to do due to a lack of appropriate measures, as high levels of incomplete data were observed using the VVIQ. This led to the development of a new mental imagery questionnaire for this population (the Mental Imagery Questionnaire for people with intellectual disabilities; MIQ‐ID). Initial testing of the psychometric properties of this measure is ongoing.

The systematic approach to intervention refinement, guided by the FRAME expanded framework, demonstrates the value of iterative development when working with specific populations. The documented adaptations illustrate the importance of modifying interventions based on participant feedback to enhance acceptability and potentially feasibility.

The flexibility highlighted by supporters as particularly valuable suggests that while manualised interventions provide important structure, maintaining adaptability to individual needs and circumstances is crucial for this population.

### 4.4 Future Research Directions

This initial testing provides a foundation for larger‐scale evaluation of Co‐MAID. Future research should include a feasibility study with larger samples. Longer follow‐up periods are needed to assess maintenance of progress and identify factors that support implementing and practising new skills.

Research is also needed to identify which participants are most likely to benefit from mental imagery interventions and to refine eligibility criteria. The role of supporter involvement requires further investigation to determine which areas of the intervention particularly benefit from supporter input.

Other areas for consideration include refining outcome measures for this population, exploring the use of technology in delivering interventions, understanding the active ingredients of the intervention and investigating the optimal intervention dosage.

## 5 Conclusion

This study provides encouraging initial evidence that mental imagery‐based interventions can be acceptable and effective for people with mild to moderate intellectual disabilities experiencing anxiety.

The co‐design process and iterative refinement demonstrate the importance of involving people with intellectual disabilities as partners in intervention development. While further research is needed to establish efficacy and optimal implementation, these findings suggest that mental imagery techniques should be considered as part of the therapeutic toolkit for supporting mental health in people with intellectual disabilities.

## Funding

This work was supported by the National Institute for Health and Care Research (NIHR300501).

## Conflicts of Interest

The authors declare no conflicts of interest.

## Supporting information


**Figure S1:** Qualitative session by session feedback form.

**Figure S2:** Overview of recruitment and participation.

**Figure S3:** What was helpful in therapy worksheet including endorsements by participants.

**Figure S4:** Comparison of daily mood recordings at baseline, intervention and follow up phases.

**Table S1:** Intervention refinements.

## Data Availability

The data that support the findings of this study are available on request from the corresponding author. The data are not publicly available due to privacy or ethical restrictions.
