# Supplementary material for: Initial Testing of a Novel, Mental Imagery‐Based Anxiety Intervention for People With Mild to Moderate Intellectual Disabilities Using a Single Case Experimental Design
Source: J Appl Res Intellect Disabil. 2026 Jun 17;39(3):e70264. doi: 10.1111/jar.70264 (PMC13274475; doi:10.1111/jar.70264)
Supplement: Supplementary file 1 — Figure S1: Qualitative session by session feedback form. [file JAR-39-e70264-s003.docx]

**Supplementary Figure 1: Qualitative session by session feedback**

Participant number:

Intervention session number:

Name of Therapist:

Date:

We want to know what you think about our sessions together.

How did you find the session today?


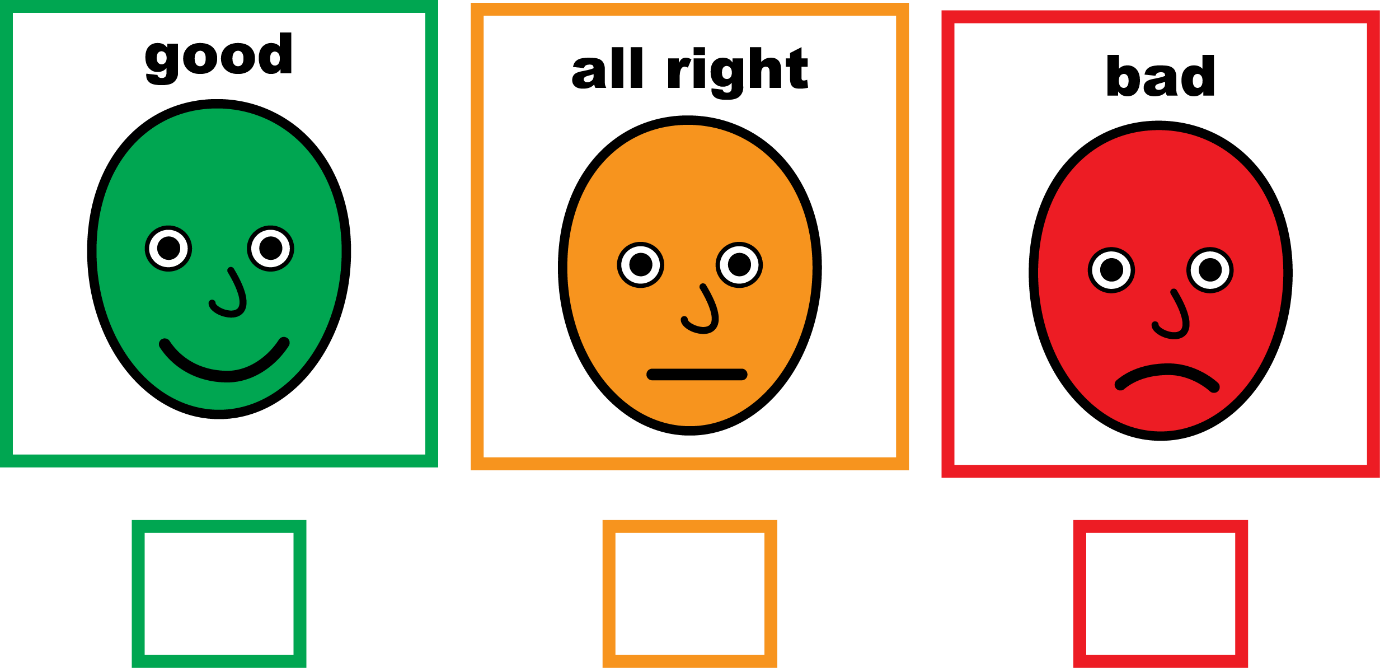


Open ended questions:

- ‘What did you like most about our time together today?’
- ‘What did you not like about the session today?’
- ‘What would you like to change about our sessions?’
- ‘Is there anything else you would like to tell me?’
