# Supplementary figures and images for: Initial Testing of a Novel, Mental Imagery‐Based Anxiety Intervention for People With Mild to Moderate Intellectual Disabilities Using a Single Case Experimental Design
Source: J Appl Res Intellect Disabil. 2026 Jun 17;39(3):e70264. doi: 10.1111/jar.70264 (PMC13274475; doi:10.1111/jar.70264)

Supplementary Figure 2: Overview of recruitment and participation


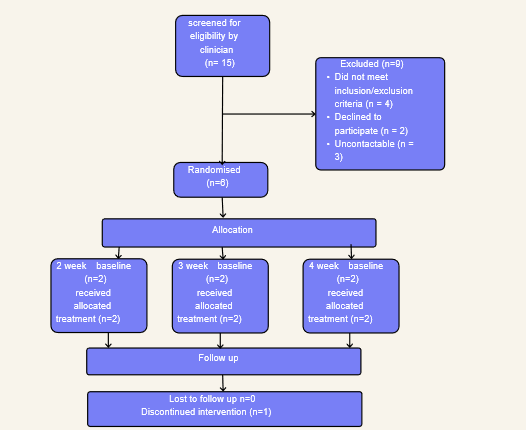

Supplement: Supplementary file 2 — Figure S2: Overview of recruitment and participation. [file JAR-39-e70264-s002.docx]
