# Supplementary material for: Initial Testing of a Novel, Mental Imagery‐Based Anxiety Intervention for People With Mild to Moderate Intellectual Disabilities Using a Single Case Experimental Design
Source: J Appl Res Intellect Disabil. 2026 Jun 17;39(3):e70264. doi: 10.1111/jar.70264 (PMC13274475; doi:10.1111/jar.70264)
Supplement: Supplementary file 3 — Figure S3: What was helpful in therapy worksheet including endorsements by participants. [file JAR-39-e70264-s001.docx]

Supplementary Figure 3: What was helpful in therapy worksheet including endorsements by participants.

| Which bits of therapy were helpful? | 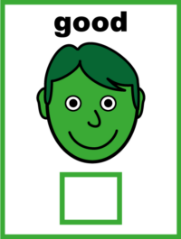 | 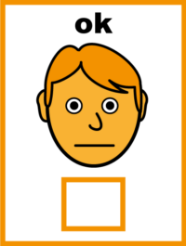 | 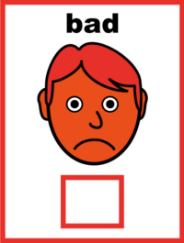 |
| --- | --- | --- | --- |
| Talking about how I feel | xxx | x | x |
|  |  |  |  |
| Getting to know my supporter better | xxx |  |  |
|  |  |  |  |
| Setting goals for therapy | xxx |  | xx |
|  |  |  |  |
| Breathing exercises | xxxx |  | x |
|  |  |  |  |
| Measuring how I feel everyday | xxxx |  | x |
|  |  |  |  |
| Doing questionnaires | xx | xxx |  |
|  |  |  |  |
| Learning about different emotions | xx | xx |  |
|  |  |  |  |
| Learning how anxiety feels in my body | xxxx | x |  |
|  |  |  |  |
| Learning that how I think changes how I feel | x | xx |  |
|  |  |  |  |
| Learning to make mental images | x | xxx |  |
|  |  |  |  |
| Doing the calm place exercise | xxx | xx |  |
|  |  |  |  |
| Doing the kind helper exercise | xxxx | x |  |
|  |  |  |  |
| Doing the switching attention exercise | xxxxx |  |  |
|  |  |  |  |
| Learning to change my mental images | xx | xx |  |
|  |  |  |  |
| Practicing new skills | xx | xx |  |
|  |  |  |  |
| Making a blueprint | xxxx |  |  |
|  |  |  |  |
| Knowing where to go if I need more help in the future | x | x |  |
|  |  |  |  |
| Something else (please write in here)   - Filling in one of the homework sheets was stressful and like schoolwork - Liked the location of sessions (not at home) - Didn’t like completing feedback forms after each therapy session - 9 sessions was not enough - Used my app to practice breathing every day - It was good to learn how to do the beach relaxation exercise [calm place] because I can use it when I am on the bus |  |  |  |
