# Supplementary material for: Initial Testing of a Novel, Mental Imagery‐Based Anxiety Intervention for People With Mild to Moderate Intellectual Disabilities Using a Single Case Experimental Design
Source: J Appl Res Intellect Disabil. 2026 Jun 17;39(3):e70264. doi: 10.1111/jar.70264 (PMC13274475; doi:10.1111/jar.70264)
Supplement: Supplementary file 4 — Figure S4: Comparison of daily mood recordings at baseline, intervention and follow up phases. [file JAR-39-e70264-s005.docx]

Supplementary Figure 4: Comparison of daily mood recordings at baseline, intervention and follow up phases

| **Participant** | **Phase comparison** | **Tau-BC** | **Standard error** | **95% CI** |
| --- | --- | --- | --- | --- |
| PPT01 | A_1_ x B | 0.47 | 0.13 | 0.19,0.67 |
| PPT01 | B x A_2_ | 0.02 | 0.14 | -0.25, 0.28 |
| PPT01 | A_1_ x A_2_ | 0.44 | 0.15 | 0.08, 0.68 |
| PPT02 | A_1_ x B | 0.22 | 0.13 | -0.08, 0.47 |
| PPT02 | B x A_2_ | -0.30 | 0.15 | -0.55, 0.01 |
| PPT02 | A_1_ x A_2_ | -0.17 | 0.19 | -0.49, 0.19 |
| PPT03 | A_1_ x B | 0.03 | 0.21 | -0.39, 0.44 |
| PPT03 | B x A_2_ | 0.16 | 0.21 | -0.25, 0.51 |
| PPT03 | A_1_ x A_2_ | 0.21 | 0.28 | -0.34, 0.64 |
| PPT04 | A_1_ x B | 0.12 | 0.10 | -0.14, 0.37 |
| PPT04 | B x A_2_ | 0.10 | 0.07 | -0.16, 0.35 |
| PPT04 | A_1_ x A_2_ | 0.22 | 0.10 | -0.09, 0.49 |
| PPT05 | A_1_ x B | 0.05 | 0.12 | -0.20, 0.30 |
| PPT05 | B x A_2_ | 0.04 | 0.12 | -0.22, 0.29 |
| PPT05 | A_1_ x A_2_ | 0.09 | 0.14 | -0.21, 0.37 |
| PPT06 | A_1_ x B | 0.39 | 0.14 | -0.01, 0.67 |
| PPT06 | B x A_2_ | missing | missing | missing |
| PPT06 | A_1_ x A_2_ | missing | missing | missing |

Table 3: Comparison of daily mood recordings at baseline, intervention and follow up phases.
