# Supplementary material for: Initial Testing of a Novel, Mental Imagery‐Based Anxiety Intervention for People With Mild to Moderate Intellectual Disabilities Using a Single Case Experimental Design
Source: J Appl Res Intellect Disabil. 2026 Jun 17;39(3):e70264. doi: 10.1111/jar.70264 (PMC13274475; doi:10.1111/jar.70264)
Supplement: Supplementary file 5 — Table S1: Intervention refinements. [file JAR-39-e70264-s004.docx]

| **Modification number** | **Who suggested modification?** | **What is to be modified?** | **Nature of the content modification** | **Reasons for modifying** | **Agreed / declined** | **Why agreed/ declined** |
| --- | --- | --- | --- | --- | --- | --- |
| 1 | OH | Print person’s workbook single sided | Change in materials | Easier to remove pages to complete homework tasks | Agreed - done 09/01/2025 |  |
| 2 | OH | Add session by session qualitative feedback form to materials for each session in person’s workbook | Change in materials | Prevent this being forgotten | Agreed – done 09/01/2025 |  |
| 3 | OH | Add adapted safe place and kind helper exercises written out in person’s workbook | Change in materials | Access to resources – allows person to refer back to exercises in written format after intervention | Agreed - Done 09/01/2025 |  |
| 4 | OH, SUP01 | Prompt (in therapist manual and person’s workbook) to check that person can open and access audio files of kind helper and safe place exercise | Change in materials | Access to resources | Agreed - Done in person’s workbook 09/01/2025 |  |
| 5 | OH | Clarify that mid intervention standardised measures should be administered after session 5 (not before) in therapist’s manual | Change in materials | Improve consistency | Declined. | Already clearly stated in therapist manual |
| 6 | OH | Supporter CRF need to add yoked participant ID and initials to form | Change in materials |  | Agreed – done 15/05/2024 |  |
| 7 | OH | Participant CRF and supporter CRF add extra boxes for ID number | Change in materials |  | Agreed – done 15/05/2024 |  |
| 8 | OH | Check consistency of language around calm/ safe place in person’s workbook, intervention protocol and therapist’s manual | Change in materials | Improve accessibility | Agreed - Done 09/01/2025 changed to ‘calm place’ |  |
| 9 | OH | Check consistency of language around kind helper in person’s workbook, intervention protocol and therapist’s manual | Change in materials | Improve accessibility | Accepted 22/1/2025 | Changed to kind helper |
| 10 | OH | Session 6 learning to move our attention page 59 has a lot of text on it – would this be better placed in therapist manual? | Change in materials | Improve accessibility | Declined – | reviewed and this level of detail is in line with the other mental imagery components. Having this detail allows people to resist the exercise post intervention. |
| 11 | OH | Need to ensure we retain a copy of ‘what was helpful in therapy worksheet’ page 71 as only copy in person’s handbook. Could add another copy for therapist to take away or continue to take photo and replicate at end of intervention | Change in materials | Tailoring/tweaking/ refining | Agreed - Done 09/01/2025  Added prompt to therapist’s manual |  |
| 12 | SUP01, PPT02, SUP03 | Change the wording on information sheet and consent from ‘interview’ to ‘chat’ | Tailoring/tweaking/ refining | Improve accessibility | Declined – | this would require ethics amendment so could change for feasibility study? |
| 13 | OH | I have tended to hold on to the person’s workbook between sessions and then hand over at the end of intervention. Should I have a spare copy which the person can keep between sessions? | Change in materials | Improving accessibility | Agreed done 15/01/2025 | Added section to therapist’s manual so participant can hold on to a copy of workbook between sessions of they want to. |
| 14 | OH | Psychoeducation materials re anxiety | Tailoring/tweaking/ refining | Information presented is too complex | Declined 21/1/2025 | In future could have a more and a less complex version of these materials – perhaps using different images (easy on the eye/ photosymbol) to reflect different levels of ability |
| 15 | OH, PPT01 | Add multi-sensory exercise to develop safe place imagery through drawing/ collage etc | Change in materials | Improve accessibility | Done 09/01/2025 | Added a specific prompt to therapist’s manual |
| 16 | OH | Have standardised assessments in more accessible online format (Microsoft forms) as well as paper options. | Change in materials | Access to resources | Declined | – to add as a change for feasibility study |
| 17 | OH, ALZ | Discard VVIQ as not sensitive to change and long and unwieldy measure. Could replace with Negative Mental Imagery Questionnaire (MIQ-N) or 3/4 separate visual analogue scales to capture change relating to different mental imagery components? | Change in materials | substituting | Agreed | Changed to MIQ-ID |
| 18 | OH, PPT02 | Should end of intervention interview be conducted by therapist or RA. Participants have found it hard to engage with someone new, especially if not familiar with intervention. | Contextual change | Improve accessibility | Declined 15/01/2025– | Ned to ensure Research Assistant for future trial has experience working with people with intellectual disabilities |
| 19 | OH, PPT02 | Should a supporter be mandatory? | Adding elements | Improve accessibility | Decline 21/01/2025 | Not a mandatory part of intervention but it is highly recommended, and a core part of intervention as reflected in our logic model |
| 20 | OH | Review inclusion / exclusion criteria. Currently very broad. Should we exclude PTSD? Additional difficulties with engagement due to sensory issues (hearing) and complex physical health issues (PPT03) | Adding elements | Reduce drop out | Declined 21/1/2025 | To revisit for feasibility trial |
| 21 | PPT02 | Assessment needs to ensure any physical health issues that may hinder engagement e.g. hearing issues are identified and addressed before starting the intervention | contextual | Physical capability | Agreed 21/1/2025 | Need to undertake comprehensive assessment to understand sensory impairments and be able to put in place strategies to support them accessing intervention. Where participant has sensory impairments that precludes them accessing materials, this would be a barrier to this intervention. |
| 22 | OH, PPT01 | Completing daily mood recordings seen as positive experience and high compliance. Could it be part of the intervention? To be added to logic model? Implications for feasibility trial when people are not required to do this going forward? | Change in materials |  | Accepted 21/1/2025 | Change to intervention – rationale changed. Participants find this daily measure helpful and is part of helping people to understand how changing their mental imagery can impact their mood.  Added to logic model |
| 23 | OH | Engagement with between session tasks is variable. Can we use apps etc to help practice skills? | Change in materials | Improving accessibility | Accepted 21/1/2025 | Apps – Olivia to look at CFT apps and see which might be useful |
| 24 | OH | Make breathing exercise available in different formats e.g. audio/ app? Some clients able to access digital information. | Change in materials | Improving accessibility | Accepted 21/1/2015 | OH to look at CFT apps and see which might be useful |
| 25 | DL | Change order of mental imagery components. Start with switching attention/ grounding, then safe place, then kind helper | In line with CFT theory | Reordering of intervention segments | Accepted 22/1/2025 | In line with CFT theory and should help participants to access session content |
| 26 | DL | Develop sensory materials (soothing touch and smell) at start of intervention (session 1 or 2) and then use at start of each session with client | In line with CFT theory | Reordering of intervention segments | Agreed 15/01/2025 | In line with CFT theory and should help participants to access session content |
| 27 | DL | Add grounding and breathing to start of every intervention session. | In line with CFT theory | Reordering of intervention segments | Agreed 15/01/2025 | In line with CFT theory and additional opportunity to practice new skills |
| 28 | SUP01 | Could set joint homework with participant and supporter working on something together | Content/ implementation | Adding elements | Agreed 21/1/2025 | Added to strategies for improving homework uptake (developed with stakeholder group) to therapist’s manual |
| 29 | SUP01 | Provide exercises (safe place/ kind helper) and blueprint as audio files | Change in materials | Improve accessibility | Declined – | this is already part of protocol |
| 30 | SUP01 | Would it be helpful if the app sent daily recordings and the attached comments as well to contextualise the scores | Change in materials | Adding elements | Declined – OH 09/01/2025 | not possible on the Daylio app and not necessary for SCED |
| 31 | PPT02 | Asthma made breathing exercises hard to engage with – could have alternative strategies? | contextual | Physical capability | Accepted 21/1/2025 | Alternatives (e.g. progressive muscle relaxation) are acceptable as long as they have the same aim/ goal |
| 32 | PPT02 | Didn’t find Rubix cube helpful but does enjoy playing games on phone – could try to incorporate things the person already enjoys or uses. | Changes in materials | Improve accessibility | Agreed - Done 09/01/2025 | prompt added to therapist’s manual |
| 33 | NHS service lead (SW) | Summary document for clinical service would be helpful to include engagement/ helpful and unhelpful strategies and recommendations for the future. Template document proposed from service | Change in materials | Improve communication with clinical services | Accepted 21/1/2025 | To complete local documents in line with NHS services. Add to therapist manual |
| 34 | OH | Ensure all mention of SUP standardised measures in therapist manual and protocol have been removed | Change in materials |  | Declined 21/1/2025 | Proxy PTOS should be collected at all time points. Feasibility trial will use proxy ratings as well as respondent measures |

Supplementary Table 1: Intervention refinements
